# Third generation autologous chondrocyte implantation is a good treatment option for athletic persons

**DOI:** 10.1007/s00167-020-06148-5

**Published:** 2020-07-15

**Authors:** Thomas Richard Niethammer, Daniel Altmann, Martin Holzgruber, Sophia Goller, Andreas Fischer, Peter Ernst Müller

**Affiliations:** 1 Department of Orthopaedics, Physical Medicine and Rehabilitation, University Hospital, LMU Munich, Marchioninistraße 15, 81377 Munich, Germany; 2 Department of Radiology, University Hospital, LMU Munich, Marchioninistraße 15, 81377 Munich, Germany

**Keywords:** Cartilage defect, ACI, Knee, Sports, Return to sport

## Abstract

**Purpose:**

Autologous chondrocyte implantation is an established method for the treatment of joint cartilage damage. However, to date it has not been established that autologous chondrocyte implantation is an appropriate procedure for cartilage defects therapy in athletic persons. The aim of this study is to analyze if third-generation autologous chondrocyte implantation is an appropriate treatment for athletic persons with full cartilage defect of the knee joints.

**Methods:**

A total of 84 patients were treated with third-generation autologous chondrocyte implantation (NOVOCART^®^ 3D). The mean follow-up time was 8 years (5–14). Sports activity was measured via UCLA Activity Score and Tegner Activity Scale before the onset of knee pain and postoperatively in an annual clinical evaluation. 41 athletic persons and 43 non-athletic persons (UCLA-Cut-off: 7; Tegner Activity Scale-Cut-off: 4) were analyzed. Patient reported outcomes were captured using IKDC subjective, KOOS, Lysholm score and VAS score on movement.

**Results:**

Patient reported outcomes (IKDC, VAS at rest, VAS on movement) showed significant improvement (*p* < 0.001) postoperatively. Athletic persons demonstrated significantly better results than non-athletic persons in the analyzed outcome scores (IKDC: *p* < 0.01, KOOS: *p* < 0.01, Lysholm score: *p* < 0.01). 96.4% of the patients were able to return to sport and over 50% returned or surpassed their preinjury sports level. The remaining patients were downgraded by a median of two points on the UCLA- and 2.5 on the Tegner Activity Scale. A shift from high-impact sports to active events and moderate or mild activities was found. Furthermore, it was shown that preoperative UCLA score and Tegner Activity Scale correlated significantly with the patient reported outcome postoperatively.

**Conclusion:**

Autologous chondrocyte implantation is a suitable treatment option for athletic persons with full-thickness cartilage defects in the knee. The return to sports activity is possible, but includes a shift from high-impact sports to less strenuous activities.

## Introduction

Autologous chondrocyte implantation (ACI) is well established as a reliable method for the treatment of joint cartilage damage. It was first described in 1994 by Brittberg et al. [6] and subsequently reported on by several other authors [25, 26] using a periosteal flap (ACI-P). Third generation ACI uses a collagen I/III scaffold instead of a periosteal flap (ACI-M) [4]. Short- to medium-term follow-ups demonstrated that the surgical procedure is effective and reliable [22, 32, 41]. Clinical results and joint function as well as defect filling in magnetic resonance imaging provide convincing results in the treatment of severe cartilage defects.

The first data from long-term studies on the third generation ACI have confirmed the promising results from the first generation ACI [1, 15, 21]. However, up to now it has not been established whether ACI is an adequate procedure for athletic persons with a high level of sports activity. Returning to the pre-injured sports level is an important goal for many of these patients [17, 28].

Return to sport and postoperative physical activity are two of the main reasons why patients decide to undergo surgery [18]. Although the resumption of sports activities in the short to medium term is well described for the first- and second-generation of ACI, to date there is a lack of data on the medium- to long-term course after third-generation ACI. The return to the preoperative sports level of the patients as a measure of success after third-generation ACI had also not been investigated. In the short term, sports activity emerged as a possible influencing and prognostic factor.

The aim of this study was to analyze, if third-generation ACI is an appropriate therapy for athletic persons with full cartilage defect of the knee joints. The importance of pre- and postoperative sports activity as a possible influencing factor in the medium to long term follow-up also needed to be evaluated. It was hypothesized that third-generation ACI is a suitable therapy option for athletic persons undergoing surgery. It was also hypothesized that both preoperative and postoperative sports activity have a positive effect on the patient reported outcome after third-generation ACI.

## Materials and methods

With an institutional review board approval from Ludwig-Maximilians-Universität München (ID 344-12), a total of 84 patients were included in the study. The follow-up time was from 5 to 14 years, with an average of 8.0 years. Patients with a full thickness cartilage defect ICRS grade III-IV of the knee joint (femoral, tibial, patella) were included. All demographic data are summarized in Table 1. Recommendations of the DGOU Clinical Tissue Regeneration Working Group [30] were used for the indication of cartilage therapy. Criteria for exclusion include axial malalignment > 5°, severe malrotation, advanced osteoarthritis > grade II, knee instability, sub-totally resected meniscus in the affected compartment meniscus and corresponding bipolar cartilage defects. The study was performed as a single-center trial and all defects were treated in the University Hospital, LMU Munich.

**Table 1:** Characteristics of the patient population (sec (second defects), MPFL (medial patella femoral ligament), HTO (high tibial osteotomy))

Characteristics	Patient cohort
Total number of patients, *n*	84
Age, years	
Mean (range)	35.2 (13–66)
Median	38.5
Sex, *n* (%)	
Male	47 (56.0)
Female	37 (44.0)
BMI, kg/m^2^	
Mean (range)	26.2 (19.0–35.3)
Median	26.0
Smokers, *n* (%)	
Smokers	22 (26.2)
Non-smokers	59 (70.2)
Unknown	3 (3.6)
Defects	
Number of lesions, *n* (%)	
One defect	68 (81)
Two defects	16 (19)
Defect size, cm^2^	
Mean (range)	5.1 (2.0–12.0) (sec.^1^ 4.5)
Median	5.0
Defect localisation, *n* (%)	
Femoral condyle (med, lat)	42 (50.0)
Patellar	37 (44.0)
Trochlea	5 (6.0)
Etiology, *n* (%)	
OCD	12 (14.3)
Trauma (< 1 year ago)	8 (9.5)
Trauma (> 1 year ago)	25 (29.8)
Unknown	39 (46.4)
ICRS Classification	III–IV
Previous surgical procedure, *n* (%)	
No	57 (67.9)
Microfracturing	13 (15.5)
Cartilage shaving	2 (2.4)
Periostal ACI	3 (3.6)
HTO	3 (3.6)
Pridie drilling	3 (3.6)
Refixation of flake	1 (1.2)
MPFL reconstruction	1 (1.2)
Osteochondral transfer	1 (1.2)
Concomitant surgery, *n* (%)	
No	52 (71.9)
MPFL reconstruction	13 (15.5)
HTO	4 (4.8)
Bone grafting	8 (9.5)
Meniscus transplant (allogene)	2 (2.4)
Anterior cruciate ligament reconstruction	5 (6.0)

### Surgical technique and rehabilitation

In an arthroscopic operation, two osteochondral cylinders (diameter 3 mm, thickness: 5–10 mm) were obtained from an unloaded zone of the knee joint. They were then sent to the manufacturer (TETEC GmbH, Reutlingen, Germany). A collagen I/III biphasic scaffold was used and the graft was completed after a cultivation time of 3–4 weeks. The cell-seeded scaffold (NOVOCART^®^ 3D) was implanted using a minimally invasive parapatellar knee arthrotomy. The cartilage defects were debrided with curettes to create a stable rim of healthy surrounding joint cartilage. Subsequently, the cartilage graft was precisely inserted into the prepared cartilage defect and finally attached to the healthy joint cartilage with resorbable suture material without fibrin glue.

Initially, patients were ordered to rest in bed for 24 h. From the second postoperative day, the affected joint was treated with CPM (Continuous Passive Movement) therapy. For femoral cartilage defects, a partial load of 20 kg was recommended for 6 weeks. In patellar cartilage defects flexion was limited for 6 weeks. During these 6 weeks, flexion was continuously increased every 2 weeks (week 1–2 Extension/Flexion 0/0/20°, week 3–4: 0/0/45°, week 5–6: 0/0/60°).

### Patient reported outcome

A questionnaire was completed preoperatively. Sports activity before knee pain was assessed. The patients were classified according to the UCLA [3] activity score and the Tegner Activity Scale [39]. Accordingly, patients were divided into two groups on the basis of the UCLA sports activity and Tegner Activity Scale before knee damage. Patients with a UCLA score of 7 and a Tegner Activity Scale of 4 or smaller were classified as “non-athletic persons”. Patients with a UCLA score larger than 7 and a Tegner Activity Scale larger than 4 were classified as “athletic persons”. 43 patients were assigned to the non-athletic persons group and 41 to the athletic persons group.

Patient related outcomes were evaluated after 12 and 24 months followed by annual evaluations. The following scores were obtained: IKDC, KOOS, Lysholm and visual analogue scale (VAS) on movement.

### Statistical analysis

The statistical program SPSS (Statistical Package for the Social Sciences, Chicago, IL, USA) was used for the statistical evaluation of the data. Prior to this study a power analysis was performed to determine the required number of patients. Powerandsamplesize.com 2013–2019 (HyLown Consulting LLC—Atlanta, GA) was used. Data from a pilot study showed an IKDC score of 64.6 (SD 21.4) for athletic persons, whereas non-athletic persons scored significantly lower with 48.5 (SD 19.2). In order to achieve a power of 80% and a level of significance *p* < 0.05, a number of patients of 25 for the group of non-athletic and 25 for the group of athletic persons was estimated. The Kolmogorov–Smirnov test was used to test for normal distribution. Depending on the test result, either the combined *t* test or the Wilcoxon test was used to identify differences between the pre- and post-operative scores of the entire patient cohort. Additional analyses tested non-athletic and athletic persons for differences in the surgical outcome. Depending on the Kolmogorov–Smirnov test either an unrelated *t* test or the Mann–Whitney-*U* test was performed. Finally, correlation analyses between UCLA-Scores respectively Tegner Activity Scale and clinical outcome parameters were performed using the Spearman-correlation.

## Results

All scores showed significant improvement (Table 2). In total, IKDC showed a significant (*p* < 0.001) increase from an average of 38.4 preoperatively to 61.6 8 years postoperatively. The results of the VAS scale also showed a significant improvement. At rest, the patients rated their pain preoperatively with an average of 3.0. At the last postoperative examination, the pain sensation was rated with 1.4 (*p* < 0.001). The pain assessment on movement also decreased from an average of 6.2 preoperatively to 3.5 8 years after third-generation ACI (*p* < 0.001).

**Table 2:** Regarding the whole patient cohort, third-generation ACI showed highly significant improvement on average 8 years after surgery in all outcome parameters assessed

	Preoperative	Postoperative	*P* value
IKDC	38.7 ± 20.8	61.6 ± 20.7	0.000
VAS at rest	3.0 ± 3.1	1.4 ± 2.0	0.000
VAS on movement	6.1 ± 3.1	3.5 ± 2.7	0.000

Microfracturing was necessary in seven cases with partial insufficiency in the border zone and a high tibial osteotomy was performed because if a complete insufficiency of the ACI graft. Because of arthrofibrosis, we performed an arthrolysis in four cases. In two cases, a diagnostic knee arthroscopy was performed because of persistent pain without intervention. In three cases, a retrograde drilling was performed because of a symptomatic bone marrow edema.

The patient groups categorized according to the UCLA and the Tegner Activity Scale were examined for interfering factors (Table 3). Subsequently, the clinical outcomes of the two groups were compared.

**Table 3:** Group-analysis and clinical outcome—athletic persons vs. non-athletic persons

	Non-athletic persons	Athletic persons	*p* value
Age, years (range)	38.2 (17–66)	32.1 (13–57)	0.021*
Side, *n*			
Right	22	19	n.s
Left	21	22	
Sex, *n*			
Male	20	27	n.s
Female	23	14	
Smoker, *n*			
Smoker	12	10	n.s
Non-smoker	31	29	
BMI, kg/cm^2^ (range)	27.5 (19.6–35.0)	24.8 (19.0–35.3)	0.002*
Number of lesions, *n*			
One defect	36	32	n.s
Two defects	7	9	
Defect localization, 1st; 2nd defect *n* (%)			
Medial femoral	21 (48.8); 1 (2.3)	16 (39.0); 3 (33.3)	n.s
Lateral femoral	2 (4.7); 0 (0)	5 (12.2); 1 (11.1)	
Patellar	19 (44.2); 2(4.7)	17 (41.5); 0 (0)	
Trochlear	1 (2.3); 4 (9.3)	3 (7.3); 4 (44.4)	
Tibial	0 (0); 0 (0)	0 (0); 1 (11.1)	
Defect-size, 1st; 2nd cm^2^ mean (range)	5.2 (2–10); 3.4 (1.5–7)	5.6 (3–12); 3.3 (0.5–5)	n.s
Etiology, *n*			
OCD	5	7	n.s
Acute trauma	3	5	
Old trauma	11	14	
Unknown	24	15	
Concomitant surgery, *n*			
Yes	15	17	n.s
No	28	24	
Complications, *n*			
Yes	10	8	n.s
No	33	33	
Follow-up, years (range)	7.7 (5–13)	8.4 (5–14)	n.s
Clinical outcome			
IKDC	54.2 ± 21.0	69.4 ± 17.4	0.001
KOOS	67.4 ± 20.2	78.9 ± 15.9	0.007
KOOS symptoms	52.1 ± 17.5	58.5 ± 12.6	n.s
KOOS pain	69.7 ± 21.2	80.0 ± 16.8	0.038
KOOS ADL	73.3 ± 22.0	86.2 ± 18.2	0.001
KOOS Sport/Rec	45.4 ± 32.5	63.5 ± 27.5	0.016
KOOS QOL	4.2 ± 2.9	58.6 ± 22.3	0.040
Lysholm Score	66.4 ± 23.0	78.3 ± 18.6	0.006
VAS on movement	4.2 ± 2.9	2.7 ± 2.3	0.019

The analysis of UCLA- and Tegner Activity Scale-Cut-off showed no significant differences in most of the patient- or defect-specific factors analyzed. This confirms very balanced patient cohorts in this regard. For all clinical outcome parameters assessed, the more athletic group showed significantly better results postoperatively. Athletic persons however were significantly younger and had a smaller BMI (*)

**Fig. 1:**
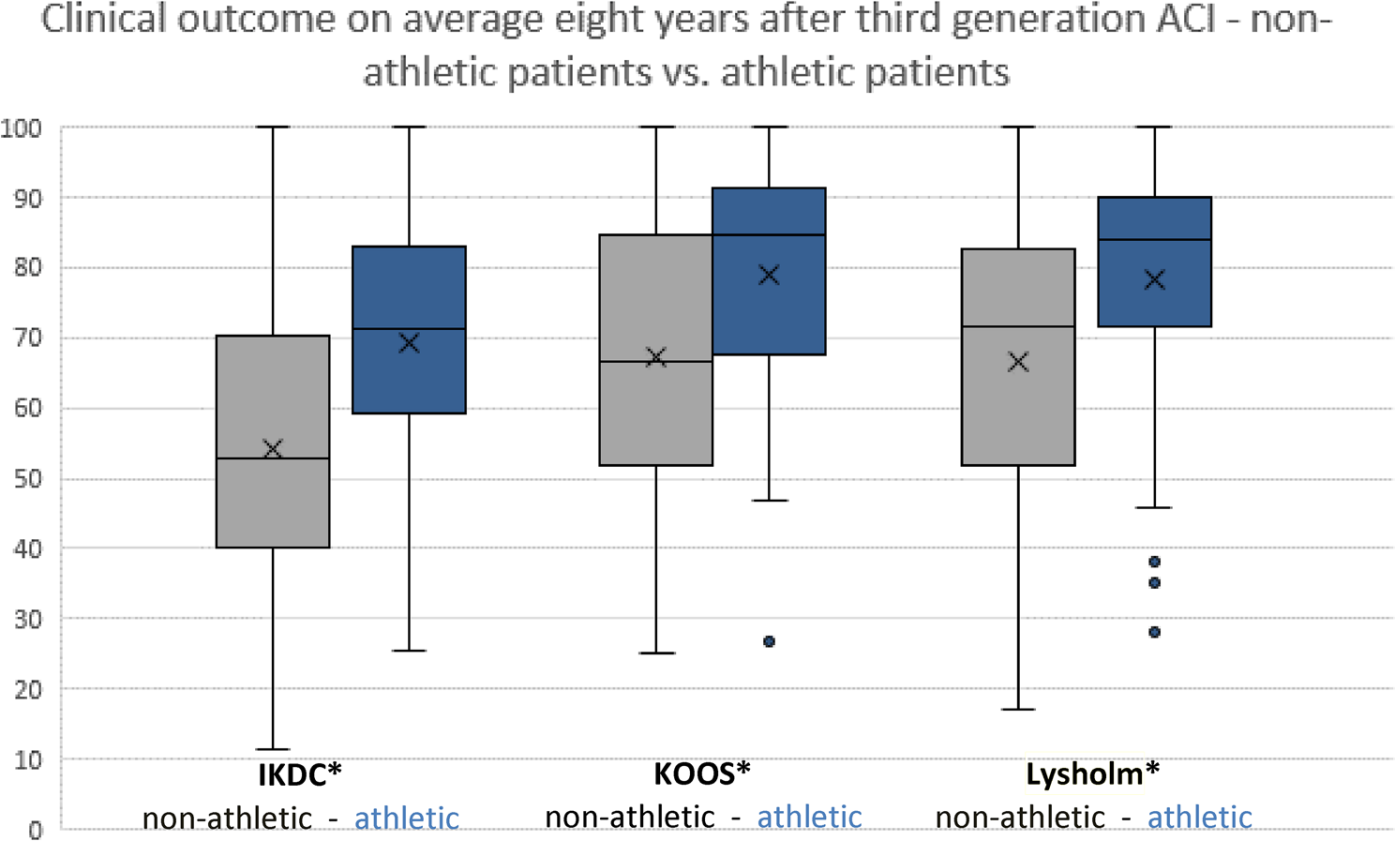
Clinical outcomes on average 8 years after third generation ACI—non-athletic persons vs. athletic persons. The outcome parameters IKDC, KOOS and Lysholm score showed significant* (*p* < 0.01) differences between the athletic persons and the non-athletic persons group

In all analyzed outcome scores, the athletic persons scored significantly better than the non-athletic persons (Table 3). In the IKDC score, non-athletic persons showed an average outcome of 54.2 points. Athletic persons showed significantly better results with 69.4 points (*p* < 0.01) (Fig. 1).

The outcome in the KOOS and in the Lysholm score also showed significant improved results. The KOOS score of the non-athletic persons differed with 67.4 from the score of the athletic persons, which reached a value of 78.9 (*p* < 0.01). Similarly, non-athletic persons showed lower Lysholm scores with 66.4 than athletic persons with 78.3 (*p* < 0.01).

**Fig. 2:**
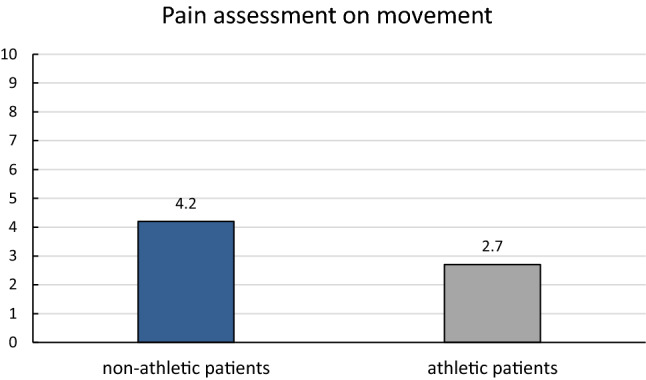
Pain assessment on average 8 years after third-generation ACI—non-athletic vs. athletic persons. Significant differences for the pain assessment on movement between non-athletic and athletic persons were present (*p* < 0.05)

In addition, the pain assessment on movement was analyzed postoperatively. Non-athletic persons assessed their pain on movement 8 years postoperatively with an average of 4.2, whereas athletic persons on movement reported only a VAS score of 2.7 (*p* < 0.05) (Fig. 2).

**Fig. 3:**
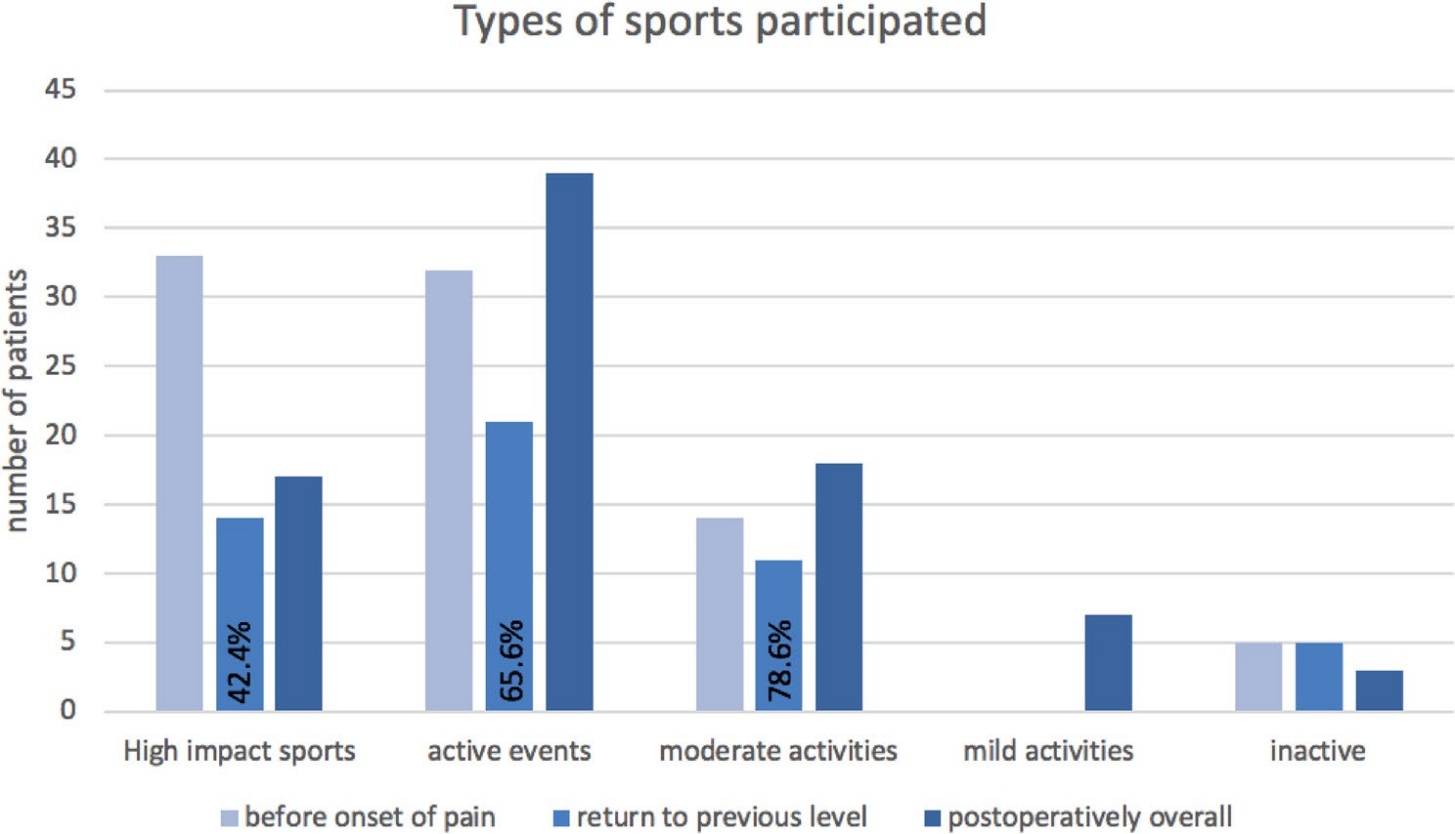
Change in types of sports participated. A shift from high-impact sports to active events and moderate or mild activities was present over the course of time

For the entire patient cohort, sports activities were documented before knee pain and postoperatively. The median sports level before knee pain was 7 for the UCLA score and 4 for the Tegner Activity Scale. Eight years postoperatively the patients still reported a median UCLA score of 7 and a median Tegner Activity Scale of 4. In total, 81 of 84 patients were able to participate in some kind of sports activity ranging from mild activities to high impact sports. This results in a return to sport rate of 96.4%. 50 patients (59.5%) were able to participate in the same type of sports postoperatively. 44 patients (52.4%) achieved at least the same UCLA-score and 48 patients (57.1%) at least the same Tegner Activity Scale postoperatively as before knee pain. Patients who did not manage to return to their level before knee pain deteriorated by a median of two points on the UCLA scale and 2.5 on the Tegner Activity Scale. The types of sports performed changed over the time. While postoperatively fewer patients performed high-impact sports than before knee pain, there was an increase in patients who performed active events and moderate or mild activities (Table 4). Only 42.4% of the patients performing high impact sports preoperatively were able to return to this level (Fig. 3).

**Table 4:** Change in types of sports participated

Types of sports	Before onset of pain	postoperatively
Inactive, *n* (%)	5 (6.0)	3 (3.6)
Mild activities, *n* (%)	0 (0.0)	7 (8.3)
Moderate activities, *n* (%)	14 (16.7)	18 (21.4)
Active events, *n* (%)	32 (38.1)	39 (46.4)
High impact sports, *n* (%)	33 (39.3)	17 (20.2)

A shift from high-impact sports to active events and moderate or mild activities was present over the course of time

Correlation analyses were carried out to analyze the influence of pre- and postoperative sports activity. The preoperative UCLA score as well as the preoperative Tegner Activity Scale correlated significantly with the patient reported outcome postoperatively. The postoperative UCLA score and Tegner Activity Scale showed significant to highly significant correlation with all patient reported outcome scores postoperatively.

## Discussion

The major finding of the study is that third-generation ACI is a suitable treatment option for athletic persons with full-thickness cartilage defects of the knee joint, with a probable return to sports activity, though often a shift from high-impact sports to active events and moderate or mild activities postoperatively. While sports activity after first- and second-generation ACI is well described, it was still not clear if ACI is a suitable procedure for athletic persons with cartilage defects.

Kreuz et al. [23] conducted a prospective study concerning the importance of sports in cartilage regeneration after autologous chondrocyte implantation. The patients were treated with ACI using a periosteal patch. The authors concluded that physical activity and in particular moderate sports are an important part of postoperative rehabilitation and should be carried out for at least 2–3 years after surgery.

Krych et al. [24] reported on return to sport after the surgical management of articular cartilage lesions in the knee on a meta-analysis basis. This meta-analysis concluded an overall return to sport rate of 76%, with highest rates after osteochondral autograft transfer (93%) followed by osteochondral allograft transplantation (88%), ACI (82%) and microfracture (58%). ACI had the highest rehabilitation time.

Kon et al. [20] conducted a prospective comparative study of arthroscopic second-generation ACI versus microfracture concerning articular cartilage treatment in 41 high-level male soccer players. They concluded that ACI may delay the return to competition, while offering more durable clinical results.

Pestka et al. [35] analyzed the return to sports activity and work after second-generation ACI. The conclusion of this study is that satisfactory results for everyday activities can be achieved and that the return to low- and moderate-intensity activities is realistic in most cases. However, the return rate to highly stressful activities was low.

Erdle et al. [14] published a study regarding sporting activity following first-generation ACI. They concluded that the premorbid level of sports and recreational activity could not be achieved 11 years after first generation ACI.

In the present study, the patient reported outcomes of “athletic persons” and “non-athletic persons” after third generation ACI were analyzed to determine if third generation ACI is a suitable treatment option for athletic persons and result in a comparable level of activity before and after knee pain. The UCLA activity score and the Tegner Activity Scale were used to classify patients according to their sporting activity. The UCLA score is a well-established and widely used score for the assessment of sports activity on a scale of 1 (“completely inactive, dependent on others and unable to leave home”) to 10 (“regularly participate in impact sports”) [11, 29]. The Tegner Activity Scale was first described in 1985 and is a highly recognized and frequently used patient-administered activity rating system for patients with various knee disorders [5, 9]. The clinical outcome of this study was measured using several well-established and widely used parameters such as IKDC [19], KOOS [10, 38] and Lysholm score [5, 9, 37] as well as visual analog scales for pain assessment [8].

The UCLA score and Tegner Activity Scale in this study showed no difference between the median before the onset of pain to eight years postoperatively. 96.4% of the patients returned to sport and more than 50% were able to even regain their preinjury sporting level. The systematic review article by Campbell et al. [7] regarding short- to medium-term return to sport showed an average rate of 84% for ACI, whereas the return to sport at preinjury level was 76%. This review included studies which varied from 31.5% [34] to 100% [23] of patients returning to their preinjury sports level. Peterson et al. [36] showed 3–5 years after ACI-P a continuation of sporting activity of 96% and Mithofer et al. [27] reported results of 52% 7 years postoperatively. These studies confirm our findings of a downward trend in sports activity in an 84 patient cohort over the medium- to long-term (8 years).

Patients who did not return to their previous sporting level postoperatively were downgraded on the UCLA scale by a median of 2 and on the Tegner Activity Scale of 2.5 points. 42.4% of the patients performing high impact sports preoperatively were able to return to this level. The reasons that this percentage is not higher are numerous: strict rehabilitation programs allow the beginning of contact sports only after more than 1 year after the surgery; recommendations from treating physicians may discourage high impact sports and suggest other active or moderate intensity activities, the fear of re-injury can inhibit patients [16], age also plays an increasingly important role [2, 13]. It is therefore possible that based on the surgery result alone, an even higher postoperative sporting level could be achieved. Similar observations have already been made in earlier studies with first-generation ACI [14].

All clinical scores showed highly significant improvements for the considered patient cohort with an average of 8 years (5–14) after third-generation ACI (*p* < 0.001). Moreover, the study demonstrated that athletic persons showed significantly better results in all clinical outcome parameters than non-athletic persons.

Correlation analyses were performed to evaluate the influence of pre- and post-operative sports activity on the medium- to long-term outcome after third-generation ACI. The UCLA score as well as the Tegner Activity Scale before knee damage showed significant results in all analyzed scores. Postoperative sports activity also showed impressive results and demonstrated significant (*p* < 0.05) correlation with all clinical outcome parameters. These results are in line with previous findings, which also reported significant correlations between pre- and postoperative sports activity and clinical outcome after ACI and microfracture [12, 14, 23]. All these results suggest that the preoperative performance level is an important indicator of the clinical outcome.

In earlier studies, the exact timing of re-entry into sporting activities was examined in detail. This was not possible in this study due to inconsistent information provided by patients. This study is limited because patients were not explicitly asked at what point in time they changed their sporting behavior and for what reasons. Earlier studies showed that the relatively rapid load build-up as well as the resumption of sport with an irritation-free knee joint is possible and recommended [23, 40]. For this reason, the patients in this study were assigned to a rehabilitation program that recommended rapid load building. The exact time of the resumption of sports could not be determined from the available documentation. Moreover, the group analysis showed that athletic persons were significantly younger and had a lower BMI than non-athletic persons. Even though the differences are small this represents a bias and further limitation of this study. However, it seems logical that younger patients often have a higher sports activity than older ones and that patients with higher sports activity have a lower BMI. Several clinical studies on ACI therapy with NOVOCART have been published in the past. They showed promising results in the medium and long term [31, 33, 41]. However, there is still a lack of randomized controlled trials that allow a final evaluation.

This study demonstrated that third-generation ACI is a suitable therapy option for athletic persons with full-thickness cartilage defects of the knee joint. Athletic persons achieved significantly better results in all outcome parameters than non-athletes. Also, pre- and post-operative sports activity levels were shown to be a very important influencing and prognostic factor for the medium- to long-term outcome after third-generation ACI. This is an important preoperative factor for the decision to use third-generation ACI, as better results can be expected in patients with higher level of sports activities.

## Conclusion

This study was able to show that third-generation ACI is a suitable therapy option for athletic persons with full-thickness cartilage defects of the knee joint. The return to sports activity is possible, and a shift from high-impact sports to less strenuous activities was observed. In addition, the study demonstrated the importance of pre- and postoperative sports activities for the medium- to long-term clinical outcome after third-generation ACI.
